# Microwave ablation for painful chest wall metastases from gastrointestinal stromal tumor: a case report

**DOI:** 10.3389/fonc.2024.1215479

**Published:** 2024-04-23

**Authors:** Shishi Wang, Lu Wang, Tingting Li, Yuan Li, Min Zhuang, Man Lu

**Affiliations:** Department of Ultrasound, Sichuan Clinical Research Center for Cancer, Sichuan Cancer Hospital & Institute, Sichuan Cancer Center, Affiliated Cancer Hospital of University of Electronic Science and Technology of China, Chengdu, China

**Keywords:** gastrointestinal stromal tumors, bone metastasis, microwave ablation, palliative care, pain management

## Abstract

**Background:**

Gastrointestinal stromal tumor (GIST) is the most common mesenchymal tumor of the digestive tract, with the potential to metastasize. Metastases to bone and soft tissue are more frequent in advanced cases, where targeted therapy is the standard treatment. However, around 10–15% of patients develop disease progression despite treatment. Studies have shown the efficacy of ablation in managing bone and soft tissue metastases (1, 2), but there are no reports of ablation for treating GIST bone or soft tissue metastases.

**Case presentation:**

In 2022, a 58-year-old man complaining of left back pain was admitted to Sichuan Cancer Hospital. He had undergone radical resection of the primary gastric GIST and vertebral metastases in 2014 and 2018, respectively. In 2019, rib metastases still occurred despite the use of targeted therapy. During the course of radiotherapy, targeted therapy, and immunotherapy, he experienced persistent chest wall pain. In addition, new lesions occurred in the lungs and chest wall in 2022. After a thorough assessment, microwave ablation (MWA) was recommended in response to his demand for immediate pain relief. The large rib metastasis constricted the spleen, so we completed the ablation in two sessions to reduce the risk of complications. He had 17 months of follow-up until September 2023, during which time his discomfort was considerably reduced.

**Conclusion:**

For GIST patients with soft tissue and bone metastases, MWA may offer substantial immediate pain alleviation. When other treatment procedures fail to achieve adequate efficacy, it provides an option.

## 1 Introduction

The most common type of mesenchymal tumor in the digestive tract is the gastrointestinal stromal tumor (GIST). Metastases occur in around 15–47% of GIST, with the liver and peritoneum being the primary locations. Metastases to the lung, bone, and soft tissue are extremely uncommon and typically observed in patients with advanced disease (3). Surgical resection is not appropriate for advanced GIST with multiple metastases. Approximately 10–15% of GIST exhibit specific gene mutations and exhibit resistance to targeted therapy (4). Radiotherapy provides analgesic effects for bone metastases, with a relief rate of 60% (5). Nevertheless, GIST is classified as a mesenchymal tumor and is known to have a limited response to chemotherapy and radiotherapy (6). Thermal ablation is an effective treatment for reducing the pain caused by bone metastases from cancer (7). However, there are currently no cases or studies on the efficacy of ablation for treating bone metastases, specifically GIST.

## 2 Case Report

**Figure 1:**
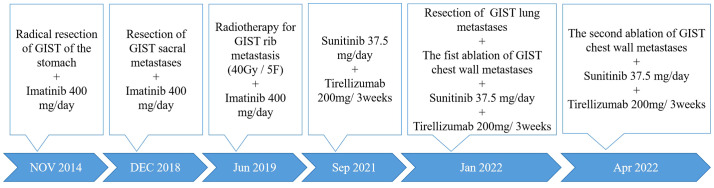
The timeline of the clinical events.

In January 2022, a 58-year-old man was admitted to Sichuan Cancer Hospital with left-sided back pain that radiated to the chest. In 2014, he was diagnosed with gastric GIST and underwent a complete surgical excision followed by targeted therapy. In 2018, he developed sacral metastases and underwent a vertebrotomy. In 2019, he experienced rib metastasis and received treatment with radiotherapy, targeted therapy, and immunotherapy. But his chest wall pain persisted, scoring a 5 out of 10 on the numerical rating scale (NRS). The timeline of the clinical events was shown in **Figure 1**.

### 2.1 Preoperative examinations

**Figure 2:**
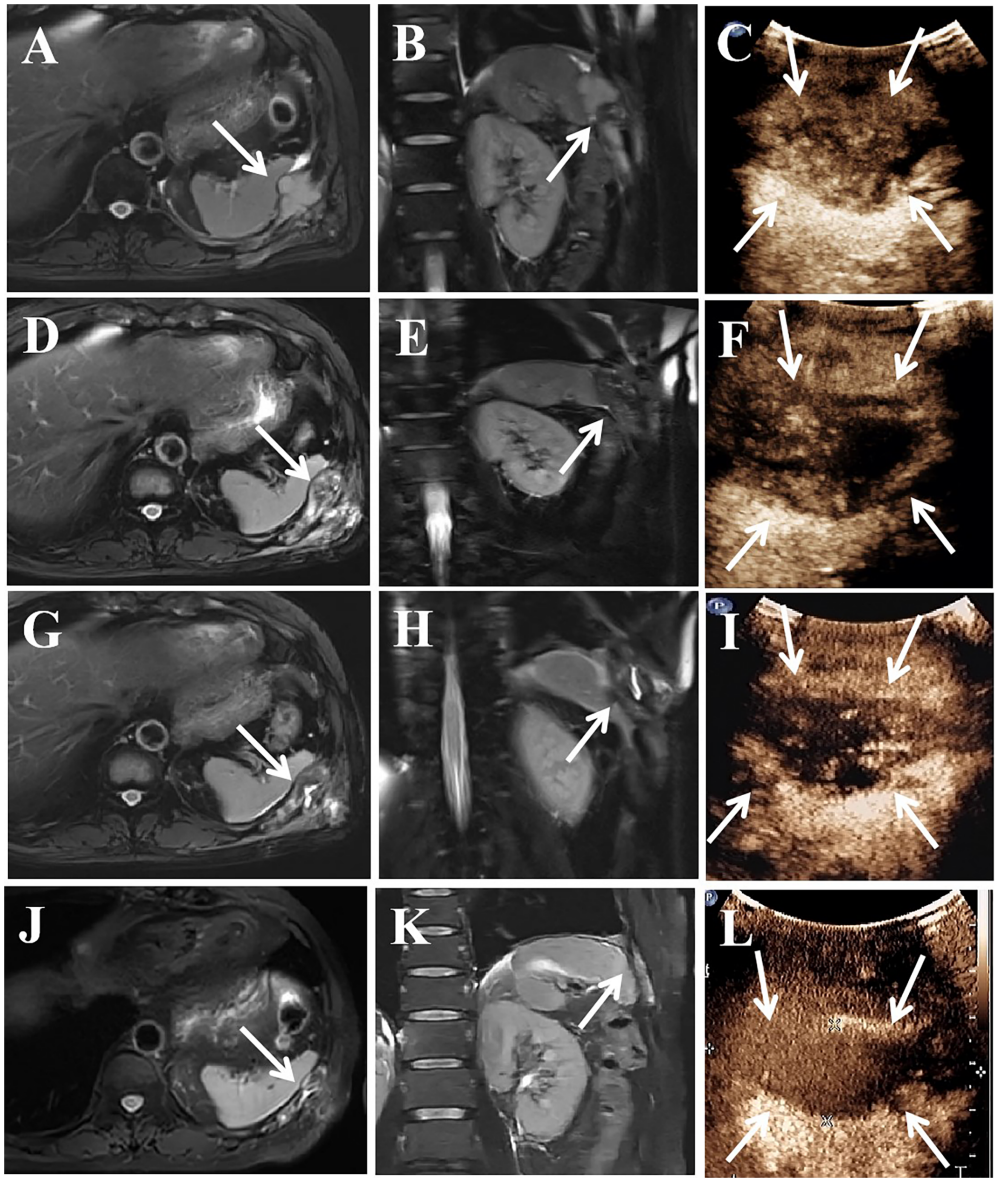
A 58-year-old man treated GIST metastases in the chest wall with MWA. **(A–C)** In January 2022, preoperative MRI showed a mass of 5.6×3.2 cm in the rib, and CEUS showed heterogeneous enhancement. **(D–F)** In February 2022, the tumor size was similar to before, CEUS showed partial enhanced area. **(G–I)** In April 2022, the tumor size was 5.3×2.4cm, and CEUS showed enhanced area. **(J–L)** 17 months after the second ablation, the tumor size was 4.7×1.6 cm, and CEUS showed no enhancement. MRI, magnetic resonance imaging; CEUS, contrast-enhanced ultrasound; the white arrow, lesion in the rib.

Magnetic resonance imaging (MRI) revealed a 5.6×3.2 cm mass in the rib, together with several solid nodules in the soft tissue (**Figures 2A, B**). Contrast-enhanced ultrasound (CEUS) showed a hypoechoic mass in the rib and several hypoechoic nodules in the chest wall, with heterogeneous enhancement in the arterial and venous phases (**Figure 2C**). Given the patient’s numerous metastases, palliative therapy was advised by the orthopedic and thoracic surgeons after consultation. Despite multiple treatment modalities, the patient continued to experience moderate pain. Consequently, microwave ablation (MWA) guided by ultrasound was advised. Postablation syndrome, characterized by fever, chills, and nausea, which is related to the size of the ablated tumor, can occur following ablation (8). Furthermore, there was a positive correlation between tumor size and complications such as thermal damage (9). Given the considerable size of the lesion and its proximity to the spleen, two separate sessions were adopted to reduce possible adverse effects.

### 2.2 Preoperative preparation

Preoperative assessments were carried out, and contraindications were ruled out. Informed consent was obtained. The diagnosis of GIST metastases was confirmed by preoperative pathological biopsies. The procedure was performed by an experienced interventional radiologist with the guidance of ultrasound (Philips EPIQ 7, Bothell, WA, USA). The ablation instrument (KY-2000; Kangyou Medical, Nanjing, China) and the microwave antenna (KY-2450B, Kangyou Medical, Nanjing, China) were used.

### 2.3 Operation process

CEUS was conducted before the ablation. 2 ml of sulfur hexafluoride lipid was administered intravenously and flushed with 5 ml of normal saline. Observe and record the volume of lesions and the vascular locations. After the safety of the puncture path was verified, the aseptic preparation was completed. 5 ml of 2% lidocaine was injected for the local anesthesia. By injecting physiological saline solution around the lesions through a 22-gauge catheter, thermal injury to normal tissues was prevented. Following the creation of a 2 mm incision in the skin at the percutaneous site, a microwave antenna was inserted into the rib lesion’s base. In January 2022, we performed the first ablation and ablated the central part of the rib lesion along with all the soft tissue lesions. The operator initiated the ablation and moved the antenna from the base to the shallow and the internal to the external, ensuring that the target lesion was covered by the vaporization area. Next, the operator ablated each lesion in the soft tissues separately. The output power was 40W, and the ablation lasted 24 minutes. After the ablation, CEUS was administered to assess the efficacy of the ablation. The absence of any enhancement in the ablation area suggests total necrosis. The pain in the left chest wall was alleviated following the first ablation, and the NRS score decreased to 2/10. In February 2022, MRI showed a comparable tumor size (**Figures 2D, E**). CEUS revealed both necrotic tissue and remaining enhanced tissue in the ablation sites (**Figure 2F**). In April 2022, a follow-up MRI scan conducted three months following the first ablation revealed that the rib lesion had decreased in size, now measuring 5.3×2.4 cm (**Figures 2G, H**). CEUS showed some degree of enhancement in the area (**Figure 2I**). The patient was in good physical condition and requested a second ablation. Therefore, we conducted further ablation to improve the local tumor control. The output power was 40W, and the ablation lasted 11 minutes. Complete ablation was confirmed by postoperative CEUS.

### 2.4 Postoperative follow-up

His follow-up period lasted for 17 months, until September 2023. The MRI and CEUS revealed that the ablation area measured 4.7×1.6 cm, which was significantly smaller (**Figures 2J–L**). The pain in the chest wall was effectively managed, and the NRS score was 2/10 during the follow-up. No adverse effects were observed in relation to the ablation.

## 3 Discussion

GIST is the prevailing mesenchyme tumor of the digestive tract. 30% of GIST patients develop metastasis after radical resection of primary tumors (10). Metastases to soft tissue and bone are uncommon and typically appear in advanced GIST. Pain relief and functional improvement are the principal objectives of bone metastases. The conventional approach for managing GIST metastasis is targeted therapy. However, primary resistance is present in 10–15% of patients, and secondary resistance develops in approximately 40–50% of patients (11). A variety of factors, including the patient’s physical condition and the number, location, and size of metastases, may impede the application of surgery. Radiotherapy provides pain alleviation for approximately 60% of patients, but it is not commonly used for GIST due to their general resistance to normal radiation doses applied to sarcomas (12). Furthermore, radiotherapy results in adverse effects such as radiation pneumonia, cardiac damage, and second malignancy, the occurrence of which is positively connected with radiation dose (13). Recent research suggest that immune checkpoints and gene mutations are associated with clinical behavior of GIST, but there is a lack of adequate clinical studies to support it (14).

MWA is a minimally invasive therapy, and studies have demonstrated its safety and efficacy in treating painful bone metastasis (15). Nevertheless, there were no case reports or studies using MWA to treat GIST bone and soft tissue metastases. We reported the case of a patient who developed metastases following surgical resection of the primary tumor and targeted therapy. Despite receiving multidisciplinary treatments, he was still experiencing unbearable pain in his chest wall and required immediate pain relief.

Coagulation necrosis and an inflammatory reaction were brought on by ablation (9, 16). As the size of the ablated tumor increased, so did the duration of ablation; additionally, complications became more likely (17). Multiple ablations may be employed for large or recurrent tumors (18). Because the tumor in this instance was big and close to the spleen, we chose to ablate a portion of it initially and then undergo another ablation after the tumor shrank. The size of the lesion had decreased to 5.3×2.4 cm three months subsequent to the initial ablation. The patient, who was in good condition, requested a second ablation. Throughout the two procedures, a liquid barrier was created, and a high-power, short-duration ablation strategy was employed to minimize thermal damage. In addition, real-time ultrasound guidance was used during the treatments to minimize harm to crucial structures. There were no adverse effects from the ablation procedure. He maintained a 17-month follow-up until September 2023. The pain in the chest wall significantly improved (2 vs. 5). The size of the lesion was decreased to 4.7×1.6 cm from its preoperative size of 5.6×3.2 cm.

### 3.1 Patient perspective

After the first ablation, the patient reported effective pain management and expressed a desire to achieve local tumor control through the second ablation. Patients discontinued nearly all of the pain medicine during the follow-up period and claimed that if the disease progressed, further ablation would be considered.

## 4 Conclusion

To our knowledge, this is the first report of the utilization of MWA for the treatment of bone and soft tissue metastases in GIST. Ablation has the potential to offer pain alleviation with little occurrence of complications and a high level of repeatability. When the tumor is large, repeated ablations may be necessary to prevent complications. Nevertheless, given this is a case report, more research is required to confirm the effectiveness of MWA on bone metastases from GIST.

## Data availability statement

The original contributions presented in the study are included in the article/supplementary material. Further inquiries can be directed to the corresponding author.

## Ethics statement

The study was approved by the Medical Ethics Committee of the Sichuan Cancer Hospital and Institute (SCCHEC-02-2016-001). Written informed consent was obtained from the individual for the publication of any potentially identifiable images or data included in this article.

## Author contributions

All authors listed have made a substantial, direct, and intellectual contribution to the work and approved it for publication. SW, MZ, ML designed the study and drafted the manuscript. LW, TL, and YL collected and analyzed the literature. SW wrote the manuscript, and ML supervised the study and critically revised the manuscript. All authors contributed to the article and approved the submitted version.
